# Effect of Digoxin Versus Bisoprolol for Heart Rate Control in Atrial Fibrillation With Heart Failure on Quality of Life: A Prospective Randomised Comparative Study

**DOI:** 10.7759/cureus.90171

**Published:** 2025-08-15

**Authors:** Muhammad Sarwar, Noor Un Nahar, Hajra Amin, Maheen Iqbal, Aymen Bader

**Affiliations:** 1 Cardiology, Punjab Institute of Cardiology, Lahore, PAK; 2 Acute Internal Medicine, Northampton General Hospital, Northampton, GBR; 3 Internal Medicine, Pakistan Kidney and Liver Institute, Lahore, PAK; 4 Internal Medicine, Allama Iqbal Teaching Hospital, Dera Ghazi Khan, PAK; 5 Medicine, Shiekh Zayed Hospital, Rahim Yar Khan, PAK

**Keywords:** atrial fib, heart failure, pharmacology, quality-of-life, randomised controlled trial

## Abstract

Introduction

Atrial fibrillation (AF) and heart failure (HF) often co-exist, exerting synergistic adverse effects on patients’ morbidity, quality of life (QOL) and mortality. This also poses a unique management challenge of heart failure in the AF population as compared to the sinus rhythm population. While beta blockers such as bisoprolol have been preferred treatment options for patients with heart failure, digoxin remains a cost-effective yet underrated alternative. However, its overall effect on QOL remains debated, especially in the South Asian population that carries a higher burden of heart failure than any other ethnicity.

Objective

The objective of this study was to compare the short-term effect of bisoprolol versus digoxin on quality of life in patients with permanent AF and concurrent HF in the South Asian population.

Methods

This single-centred prospective randomised comparative study was conducted at the outpatient department of Punjab Institute of Cardiology, Lahore, from March to September 2022. A total of 80 patients with permanent AF and established HF were enrolled and randomised in two groups to receive either digoxin (62.5-250 mcg/day) or bisoprolol (1.25-15 mg/day). The 36-Item Short Form Health Survey (SF-36) was administered at baseline and after three months to assess changes in QOL. Data were analysed using SPSS v25.0 (IBM Corp., Armonk, NY, USA), with significance at p ≤ 0.05.

Results

Both treatment groups significantly improved SF-36 QoL scores after three months (p < 0.001). However, the digoxin group reported significantly greater improvement compared to the bisoprolol group (mean QoL score: 76.68 ± 9.37 vs. 70.90 ± 8.00; p = 0.004). No serious adverse events or digoxin-related toxicities were reported in either group.

Conclusion

In patients with permanent AF and HF, digoxin resulted in a statistically significant improvement in short-term quality of life compared to bisoprolol. These findings suggest that digoxin may serve as a viable and possibly superior alternative to bisoprolol in patients with permanent AF and HF, with a potential role for digoxin as a first-line agent in select populations. It also highlights the need to re-evaluate current treatment preferences, especially in resource-limited settings. Further multicentric and multi-ethnic studies are needed to substantiate these findings and evaluate long-term clinical outcomes.

## Introduction

Atrial fibrillation (AF) and heart failure (HF) are intricately linked, often coexisting and influencing each other's progression through a complex interplay of pathophysiological mechanisms, eventually leading to poorer outcomes in terms of mortality and physical and general health [1]. According to a study in 2019, conducted in the UK, 33% of the patients diagnosed with HF had underlying AF [2]. AF is rapid and irregular ventricular contractions leading to impaired left ventricular filling and hence decreased cardiac output, contributing to HF [3]. The effects of hemodynamic alterations and renin-angiotensin-aldosterone system (RAAS) activation contribute to fibrosis of the myocardium [4]. Hence, treatment mainly focuses on rate control and restoring sinus rhythm until deemed by cardiologists to be permanent AF where rate control is preferred with secondary prophylaxis of complications [5]. Preferred drug options include beta-blockers and digoxin, alone or in combination [6]. Bisoprolol is commonly used in practice due to its vast implications in other cardiovascular diseases, compared to low-dose digoxin which has proven to be more cost-effective, but there needs to be clinical trials run regarding the effect of bisoprolol and digoxin on quality of life (QOL) [7].

The randomized controlled RATE-AF trial, published in 2020, was designed to compare the effect of low-dose digoxin versus bisoprolol in permanent AF patients with HF on QOL. It was inconclusive whether either drug was superior to the other in the short term. However in the longer run over 12 months, QOL improved with low-dose digoxin [8]. An observational study done in patients over 24 months concluded reduced QOL in patients treated with digoxin, but there have been concerns regarding poor outcomes resulting from other risk factors rather than digoxin itself, not to mention the observational design and selection bias being limitations of the study [9]. There is a significant gap in data on short-term QOL effects in patients using digoxin versus bisoprolol; also there is no local evidence from the South Asian population to interpret these questions.

This study aimed to address the evidence gap by conducting a prospective randomized comparative study to compare the short-term effects of digoxin and bisoprolol on QOL in patients with permanent AF and HF using the 36-Item Short Form Health Survey (SF-36) [10]. We hypothesized that digoxin would lead to greater improvement in QOL compared to bisoprolol.

## Materials and methods

Materials and methods

This study was a prospective randomized comparative study conducted at the Cardiology Outpatient Department, Punjab Institute of Cardiology, Lahore, from March 2022 to September 2022.

Ethical approval

This trial was approved by the Department of Research, Training and Postgraduate Medical Education, Punjab Institute of Cardiology, Lahore, under reference number RTPGME-Research-212/A.

Randomization

A total of 80 randomly selected patients fulfilling the inclusion criteria were recruited after gaining informed consent, and they were evaluated for heart rate on electrophysiography. They were further divided into two groups of 40 each and randomly allotted to one of the two groups by lottery method. In group A, patients were prescribed digoxin (dose range, 62.5-250 mcg/d) and in group B, patients were prescribed bisoprolol (dose range, 1.25-15 mg/d). Then patients were followed up in the Outpatient Department after three months, where they were asked questions from the SF-36 form. 

Inclusion and exclusion criteria

Patients aged 35-70 years, both genders, and diagnosed with permanent AF with established HF were included. Exclusion to the criteria were (i) patients with neuropsychiatric disorders (established on medical records); (ii) patients with myocardial infarction in the last six months; (iii) contraindications for trial drugs; (iv) baseline heart rate <100 bpm, second- or third-degree heart block, pacemaker dependency; (v) obstructive hypertrophic cardiomyopathy, myocarditis or pericarditis; (vi) major surgery in last three months; (vii) malignancy; and (viii) unstable and AF secondary to non-cardiac causes.

Statistical analysis

After verbal consent, patients in both treatment groups were evaluated using the SF-36 at baseline (0 months) and after three months of therapy for QOL. Data were entered and analysed using IBM SPSS Statistics for Windows, Version 25.0 (IBM Corp., Armonk, NY, USA). For quantitative variables such as age and QOL scores, mean and standard deviation (SD) were calculated. For categorical variables like age groups and gender, frequencies and percentages were reported. To compare QOL scores between the two treatment groups, the independent samples t-test was applied. A p-value ≤ 0.05 was considered statistically significant. Data were further stratified by age and gender to control for potential confounding. Post-stratification, QOL scores were again compared between the groups within each stratum using the independent samples t-test.

## Results

Gender distribution

**Figure 1:**
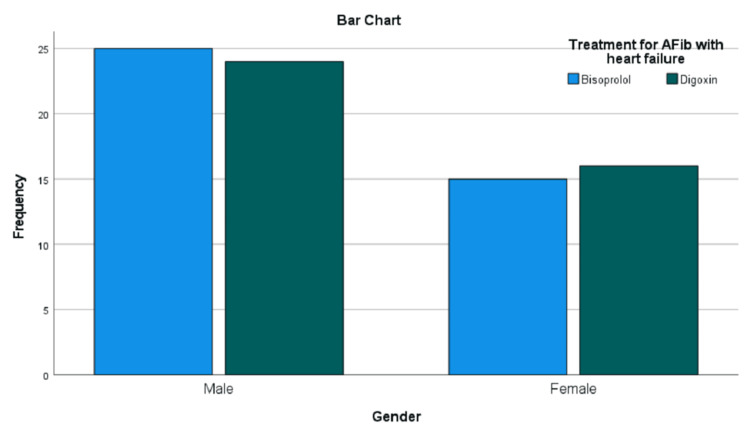
Gender distribution in digoxin vs. bisoprolol treatment groups AFib: atrial fibrillation

A total of 80 patients diagnosed with HF with AF were enrolled and evenly distributed into two treatment groups: Group A (digoxin) and Group B (bisoprolol), with 40 patients in each group. In Group A, 24 (60.0%) patients were male and 16 (40.0%) were female. In Group B, 23 (57.5%) were male and 17 (42.5%) were female. This indicates a comparable gender distribution between the two treatment groups. Figure 1 shows the distribution of both treatment groups in terms of gender.

Age distribution

**Figure 2:**
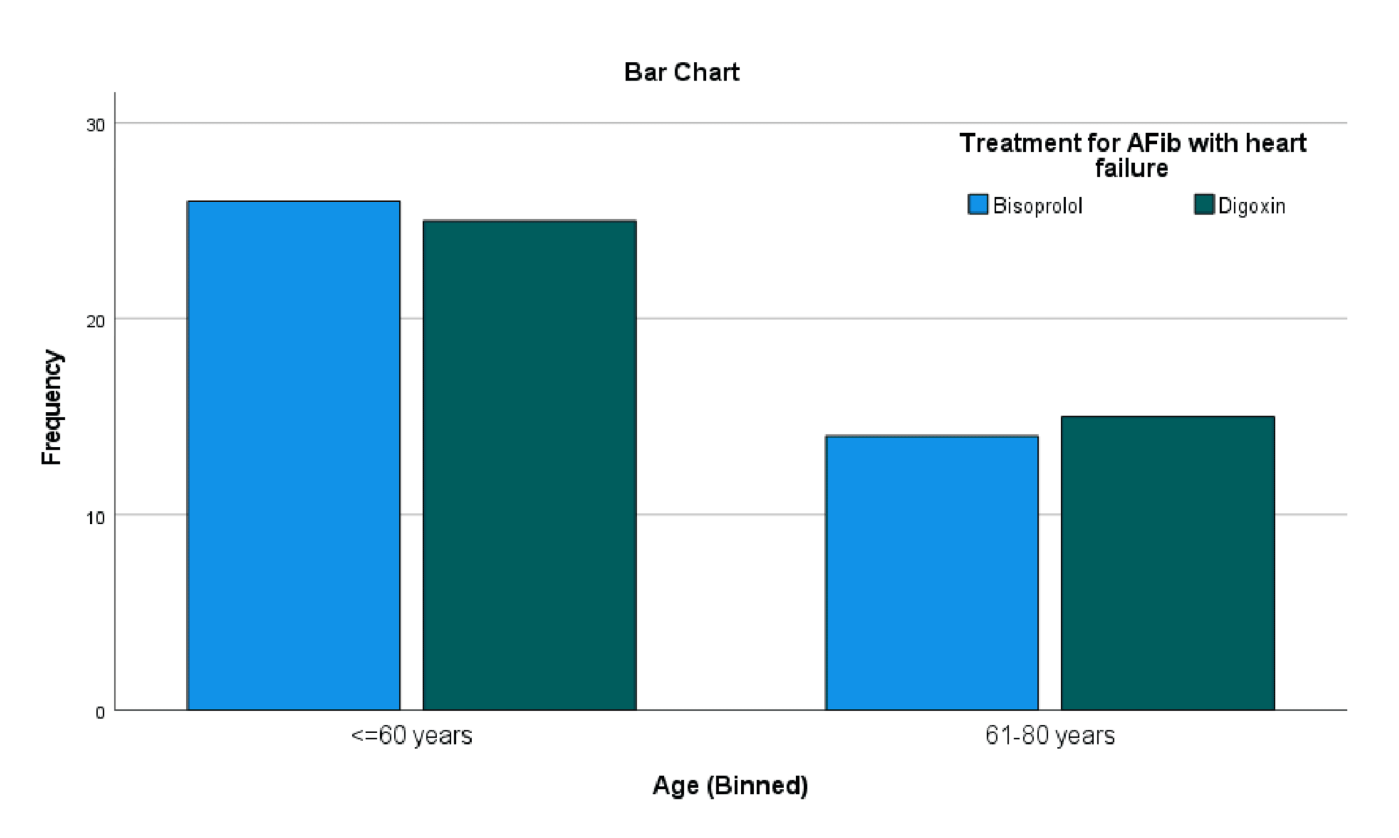
Age distribution in digoxin vs. bisoprolol treatment groups AFib: atrial fibrillation

The mean age in Group A was 55.48 ± 10.471 years, while in Group B it was 55.03 ± 10.482 years, indicating no significant difference in mean age between groups. When stratified into age categories, in Group A, 14 (35.0%) patients were aged ≤60 years and 26 (65.0%) were >60 years. Similarly, in Group B, 15 (37.5%) were ≤60 years and 25 (62.5%) were >60 years. Figure 2 reflects a balanced distribution of age among the groups.

Quality of life

**Figure 3:**
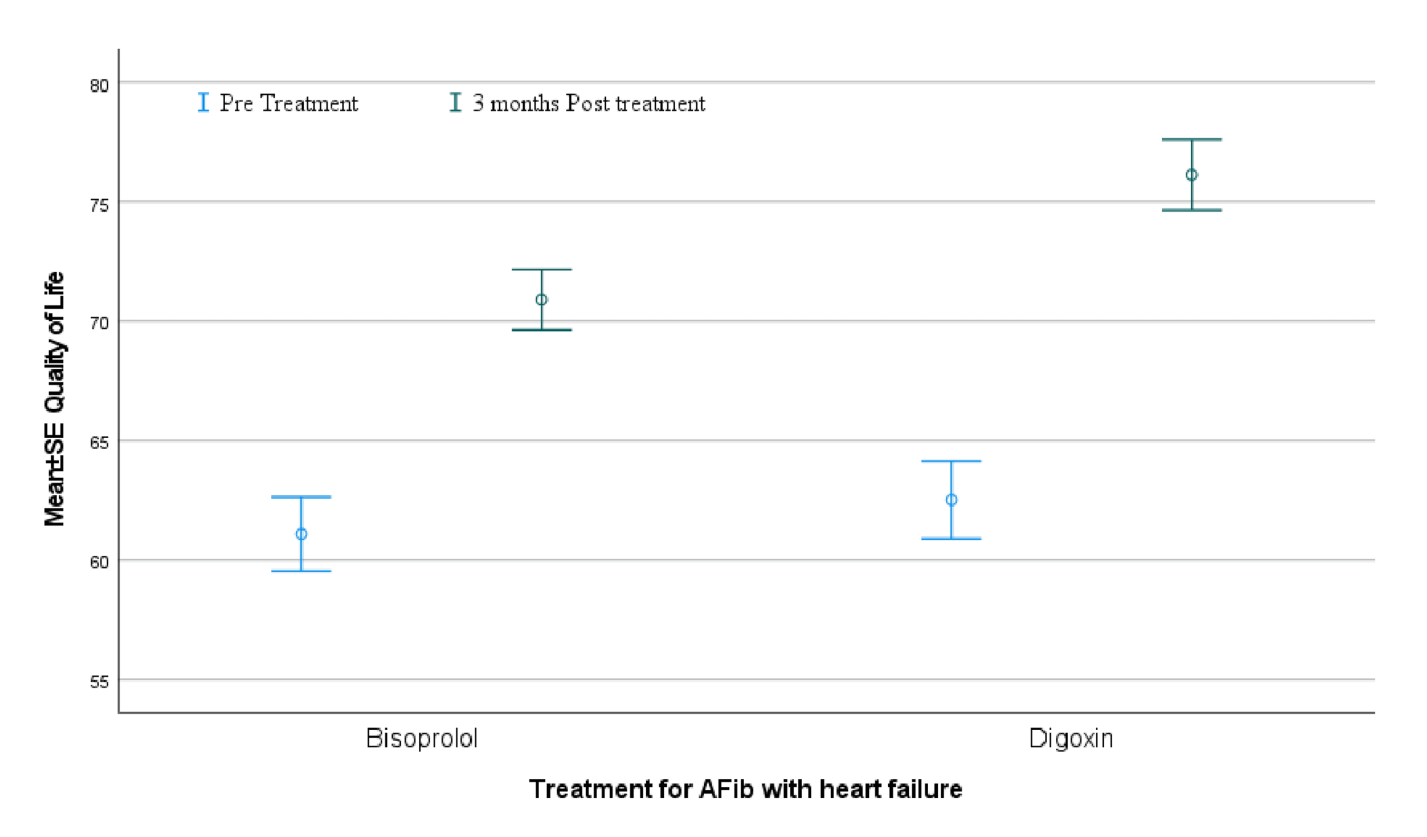
Comparison of bisoprolol vs digoxin quality of life effect at 0 (baseline) and at three months AFib: atrial fibrillation

Assessment of QOL revealed a statistically significant difference between the two treatment groups. The mean QOL score was 76.68 ± 9.37 in the digoxin group (Group A), compared to 70.90 ± 8.00 in the bisoprolol group (Group B), with a p-value of 0.009. This suggests that patients treated with digoxin reported a better-perceived QOL during the study period. Figure 3 represents visually the comparison between the two groups.

Summary of results

A total of 80 patients were analysed, with an even distribution between the bisoprolol and digoxin treatment groups. Crosstab analysis indicated no significant association between treatment type and demographic variables such as gender (χ² = 0.053, p = .818) or age (χ² = 0.054, p = .816). QOL scores significantly improved in both treatment groups after three months (bisoprolol: t(39) = -5.552, p < .001; digoxin: t(39) = -6.180, p < .001). However, the digoxin group exhibited a significantly higher post-treatment QOL score (M = 76.13) compared to the bisoprolol group (M = 70.90), with a medium effect size (Cohen’s d = 0.60; t(78) = 2.688, p = .009). These findings suggest that while both treatments are effective, digoxin may provide superior QOL outcomes in patients with AF and HF. Table 1 shows the tabular format of sig. 2-tailed p values.

**Table 1:** Comparison of quality of life scores between treatment groups using independent samples t-test

Measure	Variance Assumption	df	p-value (2-tailed)	Mean Difference
Quality of life score	Equal variances assumed	78	0.528	1.425
Quality of life score	Equal variances not assumed	77.826	0.528	1.425
Quality of life score after 3 months	Equal variances assumed	78	0.009	5.225
Quality of life score after 3 months	Equal variances not assumed	76.212	0.009	5.225

## Discussion

Currently, South Asia makes up about a quarter of the world population but disproportionately accounts for around 60% of the total burden of heart disease globally [11]. This research challenges the prevailing idea of the supremacy of bisoprolol over digoxin in patients with permanent AF and HF [7]. Traditionally, bisoprolol has been the preferred treatment modality in patients with HF. Most evidence supporting its use originates from studies conducted in the sinus rhythm population [5,6]. Hence, this management plan often overlooks the difference in treatment needs of patients with sinus rhythm and with permanent AF. Additionally, this research was conducted on a South Asian cohort, a demographic with the highest prevalence of HF both in young and elderly as compared to other ethnicities [11]. This demographic specificity adds contextual relevance to our findings [12].

Our prospective randomized comparative study compared the effect of bisoprolol and digoxin on the short-term QOL in patients with co-existing permanent AF and HF. Our findings demonstrated statistically significant improvement in QOL with digoxin as compared to bisoprolol after a three-month outpatient follow-up. This was measured by the SF-36 Questionnaire (Mean QOL; 76.68+-9.371 vs 70.90+-7.999 with a P value of 0.009). This finding supports the theory that low-dose digoxin may offer better symptomatic relief in AF patients due to its vagomimetic and neurohormonal effects [7,8].

However, these findings contradict the results previously reported in a 12-month RATE-AF trial conducted in 2020, where early results were less conclusive and pointed out there was no statistically significant difference in QOL at six months with low-dose digoxin (mean 0.161mg/day) and bisoprolol (mean dose 3.2mg/day) therapy [8]. This discrepancy may reflect the difference in study duration, endpoint or patient demographics and highlights the need for further targeted research.

These results question the widespread use of beta-blockers for HF in contrast to digoxin as a first-line therapy in patients with AF, particularly given that the current treatment regimen is extrapolated from studies in the sinus rhythm population [5,6]. Emerging real-world data also suggest comparable or even favorable clinical outcomes with digoxin in this specific population [13]. This does not underscore the unique pathophysiology and treatment needs in the context of AF and we need to re-evaluate our treatment strategy, particularly in patients with added needs.

One notable concern regarding digoxin use is its narrow therapeutic index and its potential for side effects if not monitored properly [14]. Although none of the side effects of toxicity were noted in our studies, it can be attributed to careful dosing, frequent monitoring and regular follow-ups. Even in the 2020 RATE-AF trial, despite no obvious improvement in QOL, there were significantly fewer adverse effects and lower pro-B-type natriuretic peptide (BNP) with digoxin therapy in the long term [8-15].

Limitations

Firstly, the study was conducted at a single center with a limited number of patients. Secondly, the question remains whether having other comorbidities or concomitant medication intake impacts the QOL, eventually making a significant difference or not. Thirdly, the follow-up being three months may not reflect the long-term effect of these two drug groups on QOL. Lastly, QOL is subjective depending on mood or external factors not controlled by the study. One important consideration is that our studies aimed at short-term outcomes, particularly subjective improvement in symptoms but future trials should aim to evaluate long-term outcomes including functional status, safety, pro-BNP changes and clinical endpoint (stroke, hospitalization) in a more diverse and representative population. Also, the study did not distinguish between HF with reduced or preserved ejection fraction. These remain potential areas for future investigation and there is more room to explore the gaps left here that will surely contribute to the further advancement of care.

## Conclusions

Roughly 25 years ago, digoxin was used predominantly in the treatment of HF comprising two-thirds of patients. Since then, its clinical use has markedly declined and more recently literature has paid it even less attention. It has been suggested that the cardiovascular community has dismissed digoxin as an equally acceptable treatment prematurely. Its use in HF with permanent AF should be studied particularly in different ethnicities. It can offer a viable and potentially superior QOL benefit compared to bisoprolol in selected patients with HF and permanent AF. As digoxin is readily available and affordable compared to bisoprolol, it can be used as a suitable alternative in low-resource settings.
